# Morbilliform Rash: An Uncommon Herald of SARS-CoV-2

**DOI:** 10.7759/cureus.9321

**Published:** 2020-07-21

**Authors:** Radhika B Kulkarni, Yitzchok Lederman, Agura Afiari, Jacqueline A Savage, Jason Jacob

**Affiliations:** 1 Internal Medicine, University of Connecticut, Farmington, USA; 2 Internal Medicine, University of Connecticut School of Medicine, Hartford, USA; 3 Medicine, Hartford Hospital, Hartford, USA

**Keywords:** covid-19, rash, morbilliform, sars-cov-2 (severe acute respiratory syndrome coronavirus -2), generalized rash, maculopapular rash

## Abstract

Severe acute respiratory syndrome coronavirus 2 (SARS-CoV-2) is a novel coronavirus first detected in Wuhan, China in 2019 after an outbreak of flu-like illness. The disease came to be known as the coronavirus disease of 2019 (COVID-19). It has spread quickly, spanning many countries, and has become a global pandemic. As this is a novel virus, its varied manifestations and symptomatology are coming to light daily. Although most threatening to the respiratory system, this virus has the propensity to affect multiple organ systems quickly leading to multi-organ dysfunction. Many dermatologic manifestations have been reported with no clear pattern. Most data have been anecdotal.

Here we present a 78-year-old male who tested positive for SARS-CoV-2 with no usual symptoms that would alert one of the possibilities of COVID-19. He did, however, have a diffuse morbilliform rash most notable on the trunk and back. He went on to develop fever thereafter but did not develop any respiratory complications. The rash was short-lived and was treated with topical steroids and oral antihistamines.

It is important to know and report new findings of novel diseases not only for diagnosis and treatment but also to place appropriate isolation precautions and containment. Rash may be the initial and sometimes the only manifestation of COVID-19.

## Introduction

Severe acute respiratory syndrome coronavirus 2 (SARS-CoV-2) is a novel coronavirus first described after an outbreak of flu-like illness in Wuhan, China in December 2019. The disease has been known as the coronavirus disease of 2019 (COVID-19). Respiratory complications have been a major cause of morbidity and mortality [1]. Scattered reports of a variety of skin manifestations have been associated with this disease; however, the data are limited. The prevalence of these skin manifestations has been reported as low as 0.2% in one cohort of Chinese patients and as high as 20% in an Italian cross-sectional study [1-2]. Here, we describe pruritic, morbilliform rash presenting as the initial manifestation of COVID-19.

## Case presentation

**Figure 1:**
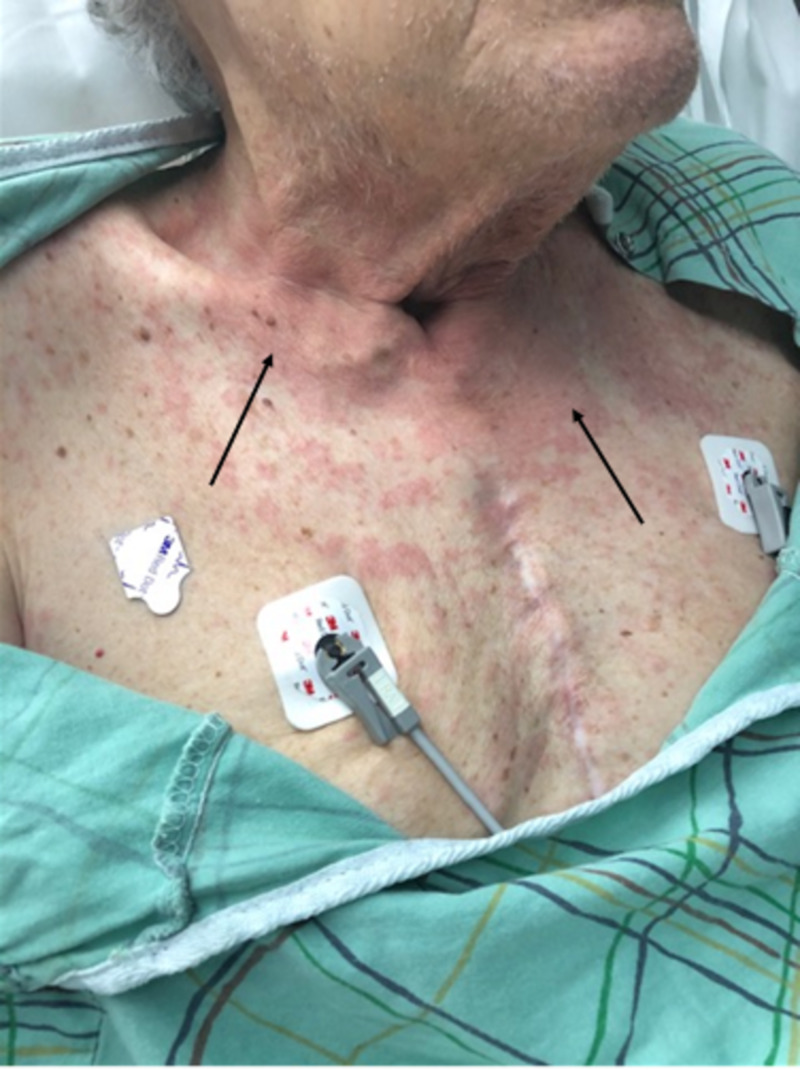
Confluent, macular rash seen predominantly on the neck and upper chest. Sternotomy scar from previous coronary artery bypass graft surgery present. Areas of confluence as indicated by black arrows.

**Figure 2:**
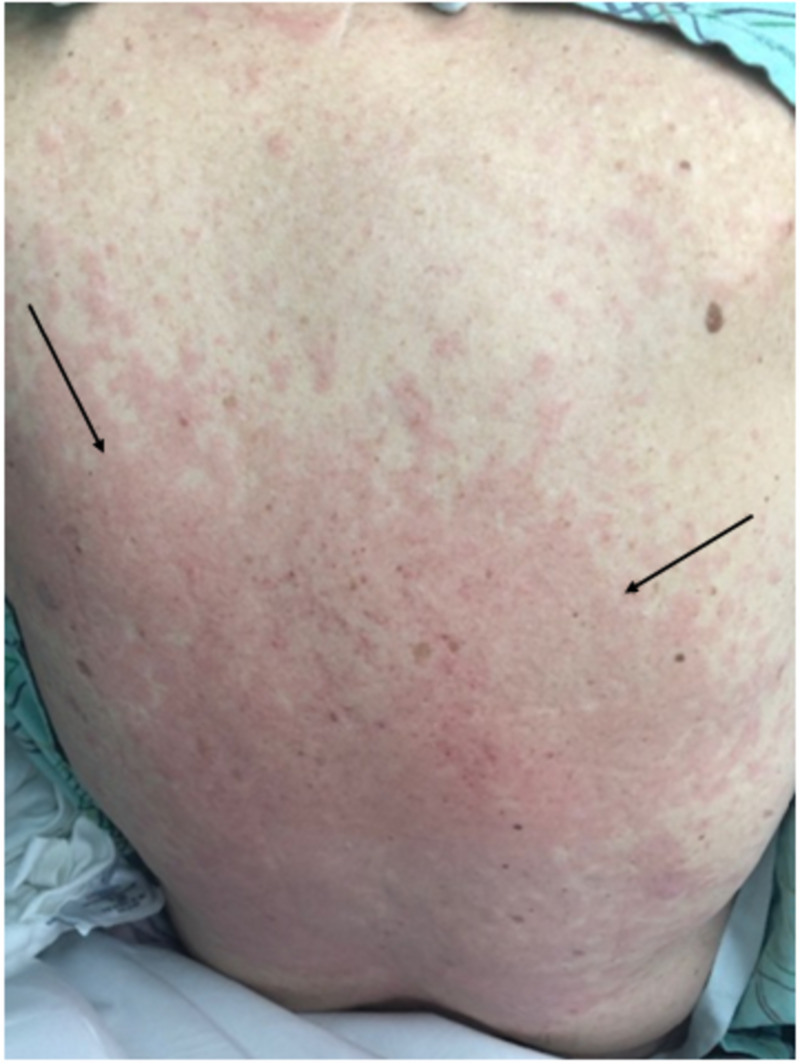
Diffuse, morbilliform rash seen on the back. Areas of confluence noted in the mid to lower part of the rash. Area of demarcation of rash and normal skin indicated by  black arrows.

A 78-year-old gentleman with a past medical history of diabetes mellitus, coronary artery disease status post coronary artery bypass graft, peripheral vascular disease, atrial fibrillation on warfarin, chronic obstructive pulmonary disease, chronic kidney disease stage 3, and bipolar disorder was brought to the ER after a mechanical fall. He denied any typical symptoms associated with SARS-CoV-2 including cough, fever, chills, shortness of breath, sore throat, or gastrointestinal symptoms. As he resided in a nursing facility, he was tested for SARS-CoV-2 (polymerase chain reaction, PCR testing) on admission which resulted positive. On physical exam, there was an erythematous, diffuse morbilliform rash without scale, most prominent on the back and the neck (Figures 1-2). The rash was maculopapular, blanchable, and with large areas of confluence. It was nontender and nonindurated on palpation. He had noticed the rash a day prior and complained of pruritis. He denied the usage of any new medications, foods, clothes, or skin products in the preceding weeks. He denied any recent travel or sick contacts. There was no significant occupational exposure as he was retired. He denied long hours of sun exposure. The patient had no other signs or symptoms of an infectious process on admission. He had no prior history of HIV, infective endocarditis, or injectable drug use. Vital signs were normal and the patient was saturating well on room air. Laboratory testing revealed a chronic normocytic, normochromic anemia (Hgb 10g/dL), mild thrombocytopenia (118,000/microliter), and chronic lymphocytopenia (absolute lymphocyte count 0.7 10*3/microliter). No abnormalities were noted on the liver panel. He had an acute kidney injury on chronic kidney disease which improved during the hospitalization. The initial chest X-ray did not show any abnormalities. A biopsy of the rash was not pursued due to the patient being on oral anticoagulants. The rash was treated with oral diphenhydramine and topical triamcinolone 0.1% cream. He later developed fever up to 101 degrees Fahrenheit, which resolved prior to discharge with no further signs of an infectious syndrome.

## Discussion

SARS-CoV-2 is a novel coronavirus first described in December 2019 in the Wuhan province of China. The classic symptoms of SARS-CoV-2 are those related to the respiratory system. A variety of skin lesions have been described in patients who tested positive for SARS-CoV-2. A case report similar to ours describes an urticarial rash as the ‘inaugural symptom’ of infection with SARS-CoV-2 before the onset of other respiratory symptoms [3]. In an Italian hospital, 148 patients who were positive for the virus were noticed to have a skin rash. Sixty of these patients were excluded as there was a history of a new medication ingested in the previous 15 days. Of the 88 patients analyzed, cutaneous manifestations observed were: erythematous rash (14 patients), widespread urticaria (three patients), and chickenpox like vesicles (one patient). These rashes mainly involved the trunk, were seldom present with pruritus, and resolved within a few days. The severity of the rash did not correlate with the severity of the systemic disease [1]. In tropical countries, certain presentations of the rash may be confused for other viral illnesses such as tick-borne diseases and dengue fever. These illnesses also present with thrombocytopenia further confounding the picture [4]. Other peculiar dermal manifestations that are not commonly associated with viral illnesses have been reported. These include transient livedo reticularis, erythematous papules resembling Chilblains disease , erythematous-yellow pruritic papules, and crusting infarcts on the tip of the toes (COVID toes) [5-8]. Acral vasculitis presenting as skin infiltrates, blisters, and superficial necrosis have been reported; these did not correspond with any known disease [9].

 Dermatologic manifestations have raised many questions as to the possible correlation with other systemic manifestations of the virus. A case series by Magro et al. described a complement-mediated process (specifically C4d and C5b-9) leading to microvascular thrombosis in both normal-appearing skin and purpuric lesions. The deposition of the aforementioned complements at alveolar capillaries was also noted on lung biopsy specimens. These studies may shed light on possible therapeutic strategies by interruption of complement-mediated pathways [10]. The implications of these dermatologic manifestations are yet to be fully understood. Knowledge is currently limited with respect to antibody response by SARS-CoV-2 and whether there is an element of antigen cross reactivity akin to sequelae of streptococcal pharyngitis [11]. Clinicians must be aware that skin rash may be the only manifestation of COVID-19 so that these cases are detected earlier, and appropriate containment is performed. 

## Conclusions

New aspects of this disease are coming to light on a daily basis. It is prudent to know and report the various presentations of COVID-19 to ensure early diagnosis and treatment. It is imperative for clinicians to be aware that skin rash may be the only manifestation of COVID-19 so that these cases are detected earlier and appropriate containment is performed. The interplay between the pathophysiology of skin rash and more dangerous systemic manifestations remains unclear. As this case is being submitted in the midst of the current COVID-19 pandemic, the information gathered is limited by its dynamic nature. Knowledge regarding the disease manifestations and treatment strategies continues to evolve.
